# Socioeconomic disparities in use of rhythm control therapies in patients with incident atrial fibrillation: A Finnish nationwide cohort study

**DOI:** 10.1016/j.ijcha.2022.101070

**Published:** 2022-06-13

**Authors:** Konsta Teppo, Jussi Jaakkola, Fausto Biancari, Olli Halminen, Miika Linna, Jari Haukka, Jukka Putaala, Pirjo Mustonen, Janne Kinnunen, Alex Luojus, Saga Itäinen-Strömberg, Juha Hartikainen, Aapo L. Aro, K.E. Juhani Airaksinen, Mika Lehto

**Affiliations:** a University of Turku, Turku, Finland; b Heart Unit, Satakunta Central Hospital, Pori, Finland; c Heart Center, Turku University Hospital, Turku, Finland; d Heart and Lung Center, Helsinki University Hospital and University of Helsinki, Helsinki, Finland; e Clinica Montevergine, GVM Care & Research, Mercogliano, Italy; f Department of Industrial Engineering and Management, Aalto University, Espoo Finland; g Aalto University, Espoo, Finland; h Neurology, Helsinki University Hospital, and University of Helsinki, Helsinki, Finland; i University of Helsinki, Helsinki, Finland; j University of Eastern Finland, Kuopio, Finland; k Heart Center, Kuopio University Hospital, Kuopio, Finland; l Lohja Hospital, Department of Internal Medicine, Lohja, Finland

**Keywords:** Atrial fibrillation, Antiarrhythmic therapies, Rhythm control therapies, Catheter ablation, Income, Socioeconomic disparities

## Abstract

**Background:**

In patients with atrial fibrillation (AF), socioeconomic disparities have been reported in the use of oral anticoagulant therapy and outcomes, but whether income also affects the utilization of antiarrhythmic therapies (AATs) for rhythm control is unknown. We assessed the hypothesis that AF patients with higher income are more likely to receive AATs.

**Methods:**

The nationwide retrospective registry based FinACAF cohort study covers all patients with AF from all levels of care in Finland. Patients were divided in AF diagnosis year and age-group specific income quintiles according to their highest annual income during 2004–2018. The primary outcome was the use of any AAT, including cardioversion, catheter ablation, and fulfilled antiarrhythmic drug (AAD) prescription.

**Results:**

We identified 188 175 patients (mean age 72.6 ± 13.0 years; 49.6% female) with incident AF during 2010–2018. Patients in higher income quintiles had consistently higher use of all AAT modalities. When compared to patients in the lowest income quintile, the adjusted incidence rate ratios (95% CI) in the highest quintile were 1.53 (1.48–1.59) for any AAT, 1.71 (1.61–1.81) for AADs, 1.43 (1.37–1.49) for cardioversion, and 2.00 (1.76–2.27) for catheter ablation. No temporal change during study period was observed in the magnitude of income disparities in AAT use, except for a decrease in income-related differences in the use of AADs.

**Conclusion:**

Profound income-related disparities exist in AAT use among patients with AF in Finland, especially in the use catheter ablation.

## Highlights

• Income-related disparities in the use of rhythm control therapies were observed.
• Differences were largest in the use of catheter ablation procedures.
• The income-related disparities did not increase during the observation period.
• These findings may signal income inequity in the provided care.

## 1 Introduction

Atrial fibrillation (AF) is the most common cardiac arrhythmia with a prevalence as high 4.1%, and it is associated with substantial mortality and morbidity, including ischemic stroke, dementia, and heart failure [1], [2], [3], [4]. AF symptoms range from none to disabling, often impairing daily life with exercise intolerance and arrhythmia-related psychological distress, thereby reducing quality of life [5]. While rate control is a reasonable treatment strategy in many patients with AF, certain aspects clearly support electing a rhythm control strategy, i.e., pursuing to restore and maintain sinus rhythm using antiarrhythmic therapies (AATs), including catheter ablation and cardioversion procedures and antiarrhythmic drugs (AADs) [3]. Rhythm control strategy has been shown to relieve symptoms and improve quality of life in symptomatic AF patients, and symptoms are the primary indication for AATs in current guidelines [3]. A recent study also suggested that early pursuit of rhythm control strategy could reduce the risk of adverse cardiovascular outcomes [6]. Furthermore, in selected patients with AF and heart failure, catheter ablation has been shown to decrease hospitalizations and mortality as well as improve functional capacity and left ventricular ejection fraction [7], [8].

Previous literature has indicated that socioeconomic inequality in health is pervasive and rising, with differences in health care financing mechanisms affecting the magnitude of health disparities [9], [10], [11], [12]. Finland, as other Nordic countries, has a universal and tax-funded health care system, full coverage of public health insurance and high reimbursement rates of medical treatment [13], [14]. Notwithstanding, socioeconomic health disparities exist in Finland in terms of somatic and psychiatric morbidity, self-rated health, and mortality [15].

In patients with AF, lower income and socioeconomic status have been associated with lower overall use of oral anticoagulant therapy and lower use of newer generation direct oral anticoagulants as well as with worse outcomes [16], [17], [18], [19]. However, evidence on the association of income level with the utilization of AATs is limited. Therefore, the present nationwide cohort study, covering all patients with AF in Finland, aimed to investigate the impact of patients’ income on the use of AATs in patients with incident AF during 2010–2018.

## 2 Methods

### 2.1 Study Population

The FinACAF Study (Finnish AntiCoagulation in Atrial Fibrillation) (ClinicalTrials Identifier: NCT04645537; ENCePP Identifier: EUPAS29845) is a retrospective nationwide registry-based cohort study including all patients with an AF diagnosis in Finland during 2004–2018 [4]. Patients were identified from three national health care registers (hospitalizations and outpatient specialist visits: HILMO; primary health care: AvoHILMO; and National Reimbursement Register upheld by Social Insurance Institute: KELA). The inclusion criterion for the cohort was an International Classification of Diseases, Tenth Revision (ICD-10) diagnosis code I48 (including atrial fibrillation and atrial flutter, together referred as AF) recorded between 2004 and 2018 and cohort entry occurred at the date of the first recorded AF diagnosis. The exclusion criteria were age < 18 years at AF diagnosis and permanent migration abroad before December 31st, 2018. The present substudy was conducted within a cohort of patients with incident AF, established in previous studies of the FinACAF cohort [20], [21], [22]. However, patients entering the cohort before the introduction of AF specific ablation codes in 2010 were excluded. Follow-up continued until death or 31st December 2018, whichever occurred first. The patient selection process is summarized in Supplementary Fig. 1.

**Fig. 1:**
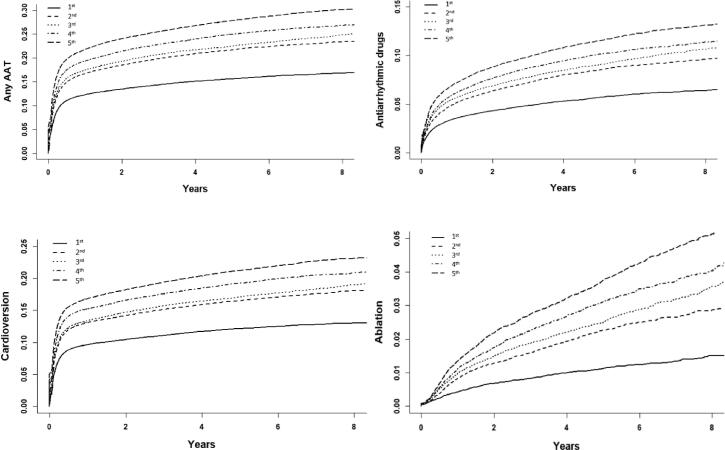
Crude cumulative incidence curves of the use of AATs according to income quintile.

### 2.2 Income

We received each patient’s highest annual taxable income (in 1000-euro accuracy) during the FinACAF study’s observation period 2004–2018 from the national Tax Register. To avoid patients’ identifiability due to high incomes, the annual income was capped to a maximum of 100 000 euros. Since income level is associated with age and the mean income level of patients may vary according to the cohort entry year, the patients were divided into age group and AF diagnosis year specific income quintiles, i.e., each 10-year age group during each cohort entry year was divided into income quintiles using age group and entry year specific cut-points [23]. Since different definitions of income levels may significantly affect the results, sensitivity analysis was performed by using cohort level income quintile cut-points [23].

### 2.3 Use of AATs

As an indicator of a pursuit of rhythm control strategy, the primary outcome was the use of any AAT, including recorded cardioversion (Nordic Classification of Surgical Procedure (NCSP) codes: TPF20, WVA50, WX904), catheter ablation (NCSP codes: TPF44, TPF45, TPF46), and fulfilled AAD prescription (ATC code C01B antiarrhythmics class I and III, plus ATC code C07AA07 sotalol). The outcome was considered to occur on the date of first fulfilled AAD prescription or procedure date after cohort entry, whichever occurred first. The secondary outcomes were cardioversion and catheter ablation procedures and fulfilled AAD prescription individually.

### 2.4 Study Ethics

The study protocol was approved by the Ethics Committee of the Medical Faculty of Helsinki University, Helsinki, Finland (nr. 15/2017) and granted research permission from the Helsinki University Hospital (HUS/46/2018). Respective permissions were obtained from the Finnish register holders (KELA 138/522/2018; THL 2101/5.05.00/2018; Population Register Centre VRK/1291/2019–3 and Tax Register VH/874/07.01.03/2019)). The patients’ identification numbers were pseudonymized, and the research group received individualized, but unidentifiable data. Informed consent was waived due to the retrospective registry nature of the study. The study conforms to the Declaration of Helsinki as revised in 2002.

### 2.5 Statistical analysis

Statistical analyses were performed with the IBM SPSS Statistics software (version 27.0, SPSS, Inc., Chicago, Illinois) and R (version 4.0.5, https://www.R-project.org). The chi-square test was used to analyze differences between proportions, and the one-way analysis of variance to compare continuous variables. Poisson regression was used to estimate incidence as well as unadjusted and adjusted incidence rate ratios (IRRs) for each AAT category and income quintile. Use of AATs may be hindered by mortality occurring during the study period, and therefore, competing risk analyses using the Fine-Gray regression model with all-cause death as a competing event were performed to estimate the unadjusted and adjusted subdistribution hazard ratios for incidence of AATs in income quintiles. In the Fine-Gray and Poisson regression models, adjustments were made for age (categorical variable in 10-year groups), gender, calendar year of AF diagnosis, education level, dementia, cancer, alcohol use disorder, psychiatric disorders, prior stroke, abnormal liver function, abnormal kidney function, diabetes, hypertension, coronary artery disease and heart failure. The definitions of the comorbidities are displayed in Supplementary Table 1.

Additionally, to assess temporal changes in the use of AATs according to income level, we determined the proportion of patients receiving AATs within one-year follow-up from cohort entry. Patients entering the cohort in 2018 were excluded from this analysis, since they had less than one year of follow-up. To statistically quantify whether the possible income-related disparities in the likelihood of receiving AATs within one year follow-up changed over study period, an interaction term between income quintile and cohort entry year as a continuous variable was fitted in a binary logistic regression model, in addition to the above stated adjusting variables.

## 3 Results

Overall, 188 175 patients (49.6% female) with incident AF in Finland during 2010–2018 were identified, and the mean age at diagnosis was 76.6 years (SD 11.6) in females and 69.3 years (SD 13.3) in males. Patients with higher income were more often male, had higher education and lower prevalence of cardiovascular comorbidities, psychiatric disorders, and alcohol abuse than patients with lower income levels (Table 1).

**Table 1:** Descriptive characteristics of the cohort according to income quintile.

**Income quintiles**	**1 (lowest)**	**2**	**3**	**4**	**5 (highest)**	**p-value**
	n = 39 348	n = 35 956	n = 37 520	n = 37 778	n = 37 573	
Mean income (thousands of euros)	2.3 (4.3)	9.9 (8.3)	16.6 (10.4)	26.8 (13.0)	57.9 (25.6)	<0.001
**Demographics**						
Mean age, years	74.2 (13.2)	72.4 (12.8)	72.9 (13.0)	72.6 (13.0)	72.6 (12.9)	<0.001
Mean cohort entry year	2014 (2.6)	2014 (2.5)	2014 (2.6)	2014 (2.6)	2014 (2.6)	<0.001
Female sex	24 954 (63.4)	20 631 (57.4)	19 554 (52.1)	16 360 (43.3)	11 821 (31.5)	<0.001
**Highest education level**						
Primary school	29 827 (75.8)	23 699 (65.9)	23 154 (61.7)	20 269 (53.7)	15 235 (40.5)	<0.001
Upper secondary education	7 983 (20.3)	9 554 (26.6)	9 998 (26.6)	9 211 (24.4)	6 300 (16.8)	<0.001
Higher education	1 538 (3.9)	2 703 (7.5)	4 368 (11.6)	8 298 (22.0)	16 038 (42.7)	<0.001
**Comorbidities**						
Abnormal liver function	274 (0.7)	208 (0.6)	182 (0.5)	171 (0.5)	163 (0.4)	<0.001
Abnormal renal function	2 034 (5.2)	1 571 (4.4)	1 663 (4.4)	1 546 (4.1)	1 449 (3.9)	<0.001
Alcohol abuse	3 184 (8.1)	1 676 (4.7)	1 264 (3.4)	1 130 (3.0)	921 (2.5)	<0.001
Cancer	7 743 (19.7)	7 181 (20.0)	7 989 (21.3)	7 984 (21.1)	8 738 (23.3)	<0.001
Coronary heart disease	9 994 (25.4)	8 308 (23.1)	8 674 (23.1)	8 473 (22.4)	7 867 (20.9)	<0.001
Dementia	2 710 (6.9)	1 930 (5.4)	1 920 (5.1)	1 816 (4.8)	1 582 (4.2)	<0.001
Diabetes	10 535 (26.8)	8 791 (24.4)	8 681 (23.1)	8 021 (21.2)	7 149 (19.0)	<0.001
Dyslipidemia	19 694 (50.1)	18 538 (51.6)	19 265 (51.3)	19 306 (51.1)	18 869 (50.2)	<0.001
Heart failure	9 105 (23.1)	6 555 (18.2)	6 312 (16.8)	5 781 (15.3)	4 800 (12.8)	<0.001
Hypertension	30 663 (77.9)	27 735 (77.1)	28 783 (76.7)	28 375 (75.1)	27 450 (73.1)	<0.001
Prior bleeding	5 037 (12.8)	4 014 (11.2)	4 190 (11.2)	4 204 (11.1)	4 045 (10.8)	<0.001
Prior ischemic stroke	5 265 (13.4)	4 119 (11.5)	4 241 (11.3)	4 001 (10.6)	3 712 (9.9)	<0.001
Prior myocardial infarction	4 112 (10.5)	3 306 (9.2)	3 368 (9.0)	3 313 (8.8)	2 835 (7.5)	<0.001
Psychiatric disorder	8 910 (22.6)	5 942 (16.5)	5 088 (13.6)	4 418 (11.7)	3 679 (9.8)	<0.001
CHA_2_DS_2_-VASc score	3.8 (1.9)	3.6 (1.9)	3.5 (1.9)	3.3 (1.9)	3.1 (1.8)	<0.001
Modified HAS-BLED score (max 8)	2.7 (1.0)	2.6 (1.0)	2.6 (1.0)	2.6 (1.0)	2.5 (1.0)	<0.001

Values denote n (%) or mean (standard deviation). Abbreviations: CHA_2_DS_2_-VASc, congestive heart failure, hypertension, age ≥ 75 years, diabetes, history of stroke or TIA, vascular disease, age 65–74 years, sex category (female); modified HAS-BLED score, hypertension, abnormal renal or liver function, prior stroke, bleeding history, age > 65 years, alcohol abuse, concomitant antiplatelet/NSAIDs (no labile INR, max score 8).

### 3.1 Use of any rhythm control therapy

During the study period, any AAT was used in 39 508 (21.0%) patients. Higher income quintile was associated consistently with higher unadjusted and adjusted incidence of any AAT use both in the Poisson and Fine-Gray regression models (Fig. 1, Table 2, Table 3). This finding was reiterated in the sensitivity analysis using cohort level income cut-points to define the income quintiles (Supplementary Table 2). Income-dependent disparities in the use of any AAT were observed across the observation period and in all age groups (Supplementary Figures 2 and 3). The differences in any AAT use between income quintiles did not change significantly during follow-up (income quintile × cohort entry year p = 0.17, Supplementary Figure 2 and Supplementary Table 3).

**Table 2:** Incidence of AATs according to income quintile.

**Outcome**	**Income quintile**	**Interventions**	**Proportion of patients with interventions**	**Patient years (in 1000 years)**	**Incidence (per 1000 patient years**	**Unadjusted IRR**	**Adjusted IRR**
Any AAT	1st	5 767	14.7%	101.8	56.7 (55.2–58.1)	(Reference)	(Reference)
	2nd	7 236	20.1%	94.6	76.5 (74.7–78.3)	1.35 (1.30–1.40)	1.18 (1.14–1.22)
	3rd	7 924	21.1%	100.0	79.2 (77.5–81.0)	1.40 (1.35–1.45)	1.25 (1.20–1.29)
	4th	8 781	23.2%	100.0	87.8 (86.0–89.7)	1.55 (1.50–1.60)	1.36 (1.31–1.41)
	5th	9 800	26.1%	98.0	100.0 (98.1–102.0)	1.77 (1.71–1.82)	1.53 (1.48–1.59)
AADs	1st	2007	5.1%	115.1	24.7 (24.4–25.1)	(Reference)	(Reference)
	2nd	2 703	7.5%	111.5	22.3 (21.6–23.0)	1.39 (1.31–1.47)	1.22 (1.15–1.29)
	3rd	3 080	8.2%	117.9	26.1 (25.2–27.1)	1.50 (1.42–1.59)	1.36 (1.28–1.44)
	4th	3 391	9.0%	120.5	28.1 (27.2–29.1)	1.62 (1.53–1.71)	1.47 (1.39–1.56)
	5th	3 885	10.3%	120.3	32.3 (31.3–33.3)	1.85 (1.76–1.96)	1.71 (1.61–1.81)
Cardioversion	1st	4 474	11.4%	106.4	42.0 (40.8–43.3)	(Reference)	(Reference)
	2nd	5 565	15.5%	101.0	55.1 (53.7–56.6)	1.31 (1.26–1.36)	1.15 (1.11–1.20)
	3rd	6 043	16.1%	107.1	56.4 (55.0–57.9)	1.34 (1.29–1.40)	1.19 (1.15–1.24)
	4th	6 822	18.1%	107.7	63.4 (61.9–64.9)	1.51 (1.45–1.57)	1.31 (1.26–1.37)
	5th	7 509	20.0%	107.2	70.0 (68.5–71.6)	1.67 (1.61–1.73)	1.43 (1.37–1.49)
Catheter ablation	1st	384	1.0%	121.3	3.2 (2.9–3.5)	(Reference)	(Reference)
	2nd	671	1.9%	119.6	5.6 (5.2–6.1)	1.77 (1.56–2.01)	1.38 (1.22–1.57)
	3rd	834	2.2%	127.0	6.6 (6.1–7.0)	2.01 (1.84–2.34)	1.60 (1.41–1.80)
	4th	997	2.6%	130.8	7.6 (7.2–8.1)	2.41 (2.14–2.71)	1.74 (1.54–1.97)
	5th	1 234	3.3%	132.3	9.3 (8.8–9.9)	2.95 (2.63–3.30)	2.00 (1.76–2.27)

Abbreviations: AAD, antiarrhythmic drug; AAT, antiarrhythmic therapy; IRR, incidence rate ratio. 95% confidence intervals in parenthesis. Unadjusted and adjusted IRRs estimated by Poisson regression and adjusted for age, sex, calendar year of AF diagnosis, education level, dementia, cancer, alcohol use disorder, psychiatric disorders, prior stroke, abnormal liver function, abnormal kidney function, diabetes, hypertension, coronary heart disease and heart failure.

**Table 3:** Risk estimates of AAT use according to the income quintile with all-cause death as a competing event.

**Outcome**	**Income quintile**	**Unadjusted SHR**	**Adjusted SHR**
Any AAT	1st	(Reference)	(Reference)
	2nd	1.42 (1.37–1.47)	1.18 (1.43–1.23)
	3rd	1.50 (1.45–1.55)	1.25 (1.21–1.30)
	4th	1.67 (1.62–1.73)	1.35 (1.30–1.40)
	5th	1.91 (1.85–1.98)	1.49 (1.44–1.55)
AADs	1st	(Reference)	(Reference)
	2nd	1.50 (1.42–1.59)	1.24 (1.17–1.31)
	3rd	1.64 (1.55–1.74)	1.38 (1.30–1.46)
	4th	1.80 (1.71–1.91)	1.50 (1.41–1.58)
	5th	2.10 (1.99–2.21)	1.71 (1.61–1.82)
Cardioversion	1st	(Reference)	(Reference)
	2nd	1.40 (1.34–1.45)	1.17 (1.12–1.21)
	3rd	1.46 (1.40–1.51)	1.21 (1.17–1.26)
	4th	1.65 (1.59–1.72)	1.32 (1.27–1.37)
	5th	1.85 (1.78.1.92)	1.42 (1.36–1.48)
Catheter ablation	1st	(Reference)	(Reference)
	2nd	1.94 (1.72–2.20)	1.40 (1.24–1.59)
	3rd	2.31 (2.04–2.60)	1.62 (1.43–1.83)
	4th	2.74 (2.43–3.08)	1.76 (1.56–2.00)
	5th	3.41 (3.04–3.82)	2.02 (1.78–2.28)

Abbreviations: AAD, antiarrhythmic drug; AAT, antiarrhythmic therapy; SHR, subdistribution hazard ratio. 95% confidence intervals in parenthesis. SHRs estimated by Fine-Gray subdistribution hazard regression and adjusted for age, sex, calendar year of AF diagnosis, education level, dementia, cancer, alcohol use disorder, psychiatric disorders, prior stroke, abnormal liver function, abnormal kidney function, diabetes, hypertension, coronary heart disease and heart failure.

### 3.2 Antiarrhythmic drugs

A total of 15 066 (8.0%) patients received AADs during the study period. The unadjusted and adjusted incidence of AAD use were higher in patients with higher income, when compared to patients in the lowest income quintile (Table 2, Table 3). Overall, use of AADs decreased over time and differences between income quintiles were observed across the study period, although some inconsistency appeared between the 2nd, 3rd, and 4th quintiles (Supplementary Figure 2). Of note, the income-related disparities in AAD use decreased significantly over the study period (income quintile × cohort entry year p < 0.001, Supplementary Table 3). When analyzing specific AADs, patients in higher income quintiles were more likely to receive flecainide, dronedarone, amiodarone and sotalol than patients in the lowest income quintile (Supplementary Table 4).

### 3.3 Cardioversions

Overall, 49 491 cardioversion procedures were performed in 30 413 (16.2%) patients. The unadjusted and adjusted rates of cardioversion were consistently higher in patients with higher income when compared to patients in the lowest income quintile (Table 2, Table 3). A similar trend was observed in the proportion of patients undergoing more than one cardioversion (Supplementary Table 4). Disparities in the performance of cardioversion between income quintiles were observed across the observation period, although the differences between the 2nd and 3rd quintiles were small and partly inconsistent (Supplementary Figure 2). The magnitude of income disparities in use of cardioversion did not change significantly over time (income quintile × cohort entry year p = 0.39, Supplementary Table 3).

### 3.4 Catheter ablations

A total of 5 021 catheter ablation procedures were performed on 4 120 (2.2%) patients during 2010–2018. The adjusted catheter ablation incidence increased steadily towards higher income quintiles (Table 2, Table 3). Likelihood of repeat ablation procedures was similarly higher in higher income quintiles (Supplementary Table 4). The overall use of catheter ablation increased steadily during 2010–2018 and income-related disparities in the use of ablation procedures were seen across the study period, although there was some variation in the annual trends during 2010–2012 (Supplementary Figure 2). No statistically significant temporal change in the magnitude of income disparities was observed (income quintile × cohort entry year p = 0.99, Supplementary Table 3).

## 4 Discussion

This nationwide cohort study demonstrated that clear income disparities exist in the use of AATs in patients with AF in Finland. Patients in higher income quintiles had consistently higher rates of use of any AAT, AADs, cardioversion and catheter ablation procedures. These income-related disparities in AAT use were observed in all age groups and across the observation period. No temporal change during study period was observed in the magnitude of income disparities in AAT use, except for the decrease in income-related differences in the use of AADs.

Previous research on the association of patients’ income and the use of rhythm control strategy in patients with AF is limited. The retrospective cohort study by Eberly et al. reported a higher rate of AAD or catheter ablation use in AF patients with a higher zip code–linked median household income [24]. However, their study covered only commercially insured patients in the United States and lacked data on patients’ individual income and education level, considerably limiting the generalizability of their results due to possible selection, information, and confounding biases. Additionally, Hagengaard et al. observed higher rate of cardioversion and catheter ablation procedures in patients with higher income, but their study included only patients hospitalized for AF with a limited follow-up of one year [25]. Similarly, a recent study conducted in Norway among patients with AF diagnosed in hospitals or specialist health care reported an association between income and higher rate of catheter ablation procedures [26]. Importantly, no study has covered all modalities of rhythm control, nor addressed the temporal trends in income-related treatment differences. Therefore, the findings of the current study, based on comprehensive data on all Finnish patients with AF from all levels of care and their individual income, substantially increase our understanding of income-related disparities in the use of rhythm control strategy in patients with AF.

The largest income-related differences were observed in the use of catheter ablation procedures, a 3-fold higher unadjusted incidence in the highest income quintile when compared to the lowest quintile. The catheter ablation incidence remained 2-fold higher even after multivariate adjustment including notably also education level. The smallest differences were observed in the use of cardioversion, wherein the highest quintile had a 67% higher crude rate of procedures compared to the lowest quintile. AATs were not used in a vast majority of patients (79%), especially among the elderly, indicating that rate control predominated as the chosen treatment approach over rhythm control strategy. Self-limiting infrequent AF episodes or asymptomatic AF patients do not generally require interventions for rhythm control, reducing the overall need of AATs in our cohort comprising of patients with all types of AF [3]. Additionally, our cohort covered uniquely also patients treated solely in primary care, hence less likely to receive AATs at all. Of note, the increasing use of catheter ablation procedures during the observation period may reflect in the observed decreasing trend in overall AAD use.

The observed differences in the utilization of AATs between income quintiles are likely multifactorial. Patients with lower socioeconomic status may have a higher threshold in seeking care unless substantial symptoms are present. Although practically all cardioversion and ablation procedures in Finland are performed in the public healthcare, more frequent use of the private sector in patients with higher income may increase prescriptions of AADs, as well as indirectly the use of cardioversion and ablation procedures through higher rate of hospital referrals. Additionally, both patient preference and advocacy for more intensive or invasive AATs may differ between income classes. Furthermore, varying levels of health literacy, differences in trust between patients and clinicians, and possible systemic biases within the health care system and society may contribute to the observed differences in treatment. Finally, the higher prevalence of cardiovascular comorbidities, dementia, and alcohol abuse disorder in lower income quintiles undoubtedly affect the clinical decision making of AATs. Nevertheless, even after adjusting for several patient characteristics, a clear disparity emerged in AAT use between income quintiles, suggesting possible inequity in the provided care.

However, our findings must be interpreted bearing in mind the several limitations of this study, especially the challenges inherent to retrospective cohort studies based on administrative data. Hence, our results represent associations and not necessarily causality between income and AAT use. Furthermore, since we lacked data on AF symptom burden, AF subclassifications and the actual reasons for withholding AATs, assumptions of lower AAT use signalling lower quality of care should be drawn with caution, especially considering the historical development in AF treatments, and that studies suggesting outcome benefits of rhythm control strategy have been published mainly in the end or after our study period [3], [6], [8]. Additionally, importantly, we lacked information on whether the patient had atrial flutter or atrial fibrillation. Although our analyses were adjusted for several patient characteristics, residual confounding cannot be excluded. Despite these limitations, the results of this large nationwide cohort study highlight important treatment differences based on income level, notwithstanding the Finnish welfare state model with universal and tax-funded health care. The findings emphasize the need for further efforts to ensure equitable access to all AF treatments. Future studies are needed to investigate the factors underlying the observed income disparities in the utilization of AATs, and in particular, whether they reflect clinically well-founded reticence or unfounded inequity in the provided care.

In conclusion, profound income-related disparities exist in the use of AATs in patients with AF in Finland, especially in the use of catheter ablation procedures. These findings are of special importance in a country that aims to ensure equity in healthcare irrespective of socioeconomic background.

## Declaration of Competing Interest

The authors declare the following financial interests/personal relationships which may be considered as potential competing interests: Konsta Teppo: none. Jussi Jaakkola: none. Fausto Biancari: none Olli Halminen: none. Jukka Putaala: Dr. Putaala reports personal fees from Boehringer-Ingelheim, personal fees and other from Bayer, grants and personal fees from BMS-Pfizer, personal fees from Portola, other from Amgen, personal fees from Herantis Pharma, personal fees from Terve Media, other from Vital Signum, personal fees from Abbott, outside the submitted work. Pirjo Mustonen: Consultant: Roche, BMS-Pfizer-alliance, Novartis Finland, Boehringer Ingelheim, MSD Finland. Jari Haukka: Consultant: Research Janssen R&D; Speaker: Bayer Finland. Miika Linna: Speaker: BMSPfizer-alliance, Bayer, Boehringer-Ingelheim. Juha Hartikainen: Research grants: The Finnish Foundation for Cardiovascular Research, EU Horizon 2020, EU FP7. Advisory Board Member: BMS-Pfizer-alliance, Novo Nordisk, Amgen. Speaker: Cardiome, Bayer. K.E. Juhani Airaksinen: Research grants: The Finnish Foundation for Cardiovascular Research; Speaker: Bayer, Pfizer and Boehringer-Ingelheim. Member in the advisory boards: Bayer, Pfizer and AstraZeneca. Mika Lehto: Consultant: BMS-Pfizer-alliance, Bayer, Boehringer-Ingelheim, and MSD; Speaker: BMS-Pfizer-alliance, Bayer, Boehringer Ingelheim, MSD, Terve Media and Orion Pharma. Research grants: Aarne Koskelo Foundation, The Finnish Foundation for Cardiovascular Research, and Helsinki and Uusimaa Hospital District research fund, Boehringer-Ingelheim. Aapo Aro: Research grants: Finnish Foundation for Cardiovascular Research; Speaker: Abbott, Johnson&Johnson, Sanofi, Bayer, Boehringer-Ingelheim.
