# Supplementary material for: Socioeconomic disparities in use of rhythm control therapies in patients with incident atrial fibrillation: A Finnish nationwide cohort study
Source: Int J Cardiol Heart Vasc. 2022 Jun 13;41:101070. doi: 10.1016/j.ijcha.2022.101070 (PMC9198807; doi:10.1016/j.ijcha.2022.101070)
Supplement: Supplementary data 1 [file mmc1.docx]

**Supplementary Material**

**Supplementary Table 1.** Definitions of the comorbidities

**Supplementary Table 2.** Sensitivity analysis of incidence of AATs using cohort level income cut-points to define income quintiles

**Supplementary Table 3.** Association of interaction terms between cohort entry year and income quintile on use of AATs within 1-year follow-up from diagnosis of AF in binary logistic regression

**Supplementary Table 4.** Use of rate control drugs, AADs and repeat AAT procedures during follow-up

**Supplementary Figure 1.** Flow-chart of the patient selection process

**Supplementary Figure 2.** Proportions of patients receiving AATs within one year follow-up according to the year of AF diagnosis

**Supplementary Figure 3.** Proportion of patients receiving any AAT within one year follow-up according to age at the time of AF diagnosis.

**Supplementary Table 1**. Definitions of the comorbidities

|  | ICD-10 | ICPC-2 | Reimbursement code | ATC code | Other |
| --- | --- | --- | --- | --- | --- |
| Hypertension | I10-I15 | K85, K86, K87 | 205 | C03A, C03B, C03DB, C03EA, C07A, C08CA, C08D, C09 |  |
| Dyslipidemia | E78 | T93 | 206 | C10 |  |
| Heart failure | I50, I11.0, I13.0, I13.2 | K77 | 201 |  |  |
| Diabetes | E10-E14 | T89, T90 | 103, 215 | A10 |  |
| Previous stroke | I63, I64, I69.3-I69.8 | K90 |  |  |  |
| Bleeding history | D50.0, D62, D68.3, I60-I62, I69.0-I69.2, I85.0, I86.4, J94.2, K22.1, K22.3, K22.6, K25.0, K25.2, K25.4, K25.6, K26.0, K26.2, K26.4, K26.6, K27.0, K27.2, K27.4, K27.6, K28.0, K28.2, K28.4, K28.6, K29.0, K62.5, K63.1, K63.3, K92.0-K92.2, N02, R04, R31, R58, S06.2-S06.6, S06.8 |  |  |  |  |
| Alcohol abuse | F10 |  |  |  |  |
| Renal failure or dialysis | N18, Z49 |  |  |  |  |
| Liver cirrhosis or failure | K70.2-K70.4, K71.7, K71.8, K72, K74 |  |  |  |  |
| Dementia | F00-F03, G30 |  |  |  |  |
| Cancer |  |  |  |  | Any cancer registered in the Finnish Cancer Registry |
| Coronary heart disease | I21-I25 |  |  |  |  |
| Prior myocardial infarction | I21-I22 |  |  |  |  |
| Psychiatric disorder | F04-F99 |  |  |  |  |

Abbreviations: ATC, anatomic therapeutic chemical; ICD-10, International Classification of Diseases, Tenth Revision; ICPC-2, International Classification of Primary Care, Second Edition

**Supplementary Table 2.** Sensitivity analysis of incidence of AATs according to income quintile when using cohort level income cut-points.

| **Outcome** | **Income quintiles** | **Incidence (per 1000 p-years)** | **Unadjusted IRR** | **Adjusted IRR** |
| --- | --- | --- | --- | --- |
| Any AAT | 1st | 38.5 (37.2-39.8) | (Reference) | (Reference) |
|  | 2nd | 52.4 (51.1-53.8) | 1.36 (1.31-1.42) | 1.18 (1.13-1.23) |
|  | 3rd | 72.5 (70.8-74.1) | 1.88 (1.81-1.96) | 1.39 (1.34-1.45) |
|  | 4th | 106.4 (104.3-108.5) | 2.76 (2.66-2.87) | 1.55 (1.49-1.62) |
|  | 5th | 135.9 (133.6-138.3) | 3.53 (3.40-3.67) | 1.79 (1.72-1.87) |
| AADs | 1st | 11.5 (10.9-12.2) | (Reference) | (Reference) |
|  | 2nd | 16.0 (15.3-16.7) | 1.39 (1.29-1.49) | 1.25 (1.17-1.35) |
|  | 3rd | 22.3 (21.4-23.1) | 1.93 (1.80-2.07) | 1.54 (1.43-1.65) |
|  | 4th | 34.4 (33.4-35.5) | 2.98 (2.80-3.19) | 1.85 (1.72-1.98) |
|  | 5th | 43.2 (42.1-44.4) | 3.75 (3.52-4.00) | 2.21 (2.05-2.37) |
| Cardioversion | 1st | 29.0 (27.9-30.1) | (Reference) | (Reference) |
|  | 2nd | 38.9 (37.8-40.0) | 1.34 (1.28-1.41) | 1.16 (1.11-1.22) |
|  | 3rd | 53.1 (51.8-54.5) | 1.83 (1.75-1.92) | 1.36 (1.30-1.43) |
|  | 4th | 73.9 (72.2-75.5) | 2.55 (2.44-2.66) | 1.47 (1.40-1.54) |
|  | 5th | 92.7 (90.9-94.5) | 3.20 (3.07-3.34) | 1.65 (1.57-1.73) |
| Catheter ablation | 1st | 1.3 (1.1-1.5) | (Reference) | (Reference) |
|  | 2nd | 2.0 (1.8-2.3) | 1.63 (1.32-2.00) | 1.24 (1.00-1.52) |
|  | 3rd | 4.0 (3.7-4.4) | 3.22 (2.65-3.90) | 1.82 (1.50-2.21) |
|  | 4th | 9.6 (9.1-10.2) | 7.63 (6.37-9.14) | 2.54 (2.10-3.06) |
|  | 5th | 14.6 (14.0-15.2) | 11.60 (9.72-13.84) | 3.17 (2.62-3.83) |
| Abbreviations: AAD, antiarrhythmic drug; AAT, antiarrhythmic therapy; IRR, incidence rate ratio. 95% confidence intervals in parenthesis. Unadjusted and adjusted IRRs estimated by Poisson regression and adjusted for age, sex, calendar year of AF diagnosis, education level, dementia, cancer, alcohol use disorder, psychiatric disorders, prior stroke, abnormal liver function, abnormal kidney function, diabetes, hypertension, coronary heart disease and heart failure. | | | | |

**Supplementary Table 3.** Association of interaction terms between cohort entry year and income quintile on use of AATs within 1-year follow-up from AF diagnosis in binary logistic regression

| **Outcome** | **Interaction term** | **OR (95% CI)** | **p-value** |
| --- | --- | --- | --- |
| Any AAT | Cohort entry year x Income quintiles |  | 0.17 |
|  | Cohort entry year x 1^st^ income quintile | (reference) |  |
|  | Cohort entry year x 2^nd^ income quintile | 1.00 (0.98-1.02) | 0.89 |
|  | Cohort entry year x 3^rd^ income quintile | 1.01 (0.99-1.03) | 0.24 |
|  | Cohort entry year x 4^th^ income quintile | 0.99 (0.97.1.01) | 0.40 |
|  | Cohort entry year x 5^th^ income quintile | 0.99 (0.97.1.01) | 0.36 |
| AADs | Cohort entry year x Income quintiles |  | <0.001 |
|  | Cohort entry year x 1^st^ income quintile | (reference) |  |
|  | Cohort entry year x 2^nd^ income quintile | 0.97 (0.94-1.00) | 0.08 |
|  | Cohort entry year x 3^rd^ income quintile | 0.99 (0.96-1.02) | 0.46 |
|  | Cohort entry year x 4^th^ income quintile | 0.96 (0.93.0.99) | 0.01 |
|  | Cohort entry year x 5^th^ income quintile | 0.94 (0.91-0.97) | <0.001 |
| Cardioversion | Cohort entry year x Income quintiles |  | 0.39 |
|  | Cohort entry year x 1^st^ income quintile | (reference) |  |
|  | Cohort entry year x 2^nd^ income quintile | 1.00 (0.98-1.03) | 0.74 |
|  | Cohort entry year x 3^rd^ income quintile | 1.02 (1.00-1.04) | 0.13 |
|  | Cohort entry year x 4^th^ income quintile | 1.00 (0.98-1.02) | 0.92 |
|  | Cohort entry year x 5^th^ income quintile | 1.01 (0.99-1.03) | 0.35 |
| Catheter ablation | Cohort entry year x Income quintiles |  | 0.99 |
|  | Cohort entry year x 1^st^ income quintile | (reference) |  |
|  | Cohort entry year x 2^nd^ income quintile | 1.00 (0.90-1.11 | 0.99 |
|  | Cohort entry year x 3^rd^ income quintile | 1.02 (0.92-1.12) | 0.77 |
|  | Cohort entry year x 4^th^ income quintile | 1.01 (0.91-1.11) | 0.91 |
|  | Cohort entry year x 5^th^ income quintile | 1.02 (0.92-1.12) | 0.75 |
| Abbreviations: AAD, antiarrhythmic drug; AAT, antiarrhythmic therapy; OR, odds ratio. ORs estimated with binary logistic regression model including age, gender, cohort entry year, education level, income level, dementia, cancer, alcohol use disorder, psychiatric disorders, prior stroke, abnormal liver function, abnormal kidney function, diabetes, hypertension, coronary heart disease and heart failure. OR<1 indicates decreasing and OR>1 increasing difference over time in the likelihood of event within 1-year follow-up compared to the lowest income quintile. | | | |

**Supplementary Table 4.** Use of rate control drugs, AADs and repeat AAT procedures during follow-up

| **Income quintiles** | **1 (lowest)** | **2** | **3** | **4** | **5 (highest)** | **P-value** |
| --- | --- | --- | --- | --- | --- | --- |
| Beta-blockers | 31 164 (79.2%) | 29 710 (82.6%) | 30 607 (81.6%) | 30 871 (81.7%) | 30 196 (80.4%) | <0.001 |
| Digoxin | 6 429 (16.3%) | 5 415 (15.1% | 5 194 (13.8%) | 5 095 (13.5%) | 4 494 (12.0%) | <0.001 |
| NDCCB | 1 200 (3.0%) | 1 140 (3.2%) | 1 133 (3.0%) | 1 137 (3.0%) | 1 017 (2.7%) | <0.001 |
| Amiodarone | 836 (2.1%) | 936 (2.6%) | 1 030 (2.7%) | 1 063 (2.8%) | 1 140 (3.0%) | <0.001 |
| Dronedarone | 98 (0.2%) | 133 (0.4%) | 165 (0.4%) | 189 (0.5%) | 176 (0.5%) | <0.001 |
| Flecainide | 877 (2.2%) | 1 406 (3.9%) | 1 621 (4.3%) | 1 835 (4.9%) | 2 110 (5.6%) | <0.001 |
| Sotalol | 185 (0.5%) | 210 (0.6%) | 248 (0.7%) | 281 (0.7%) | 428 (1.1%) | <0.001 |
| Ablations >1 | 53 (0.1%) | 135 (0.4%) | 147 (0.4%) | 181 (0.5%) | 237 (0.6%) | <0.001 |
| Cardioversions > 1 | 1 142 (2.9%) | 1 550 (4.3%) | 1 738 (4.6%) | 2 046 (5.4%) | 2 434 (6.5%) | <0.001 |
| NDCB, Non-dihydropyridine calcium channel blockers. | | | | | |  |

**Supplementary Figure 1.** Flow-chart of the patient selection process


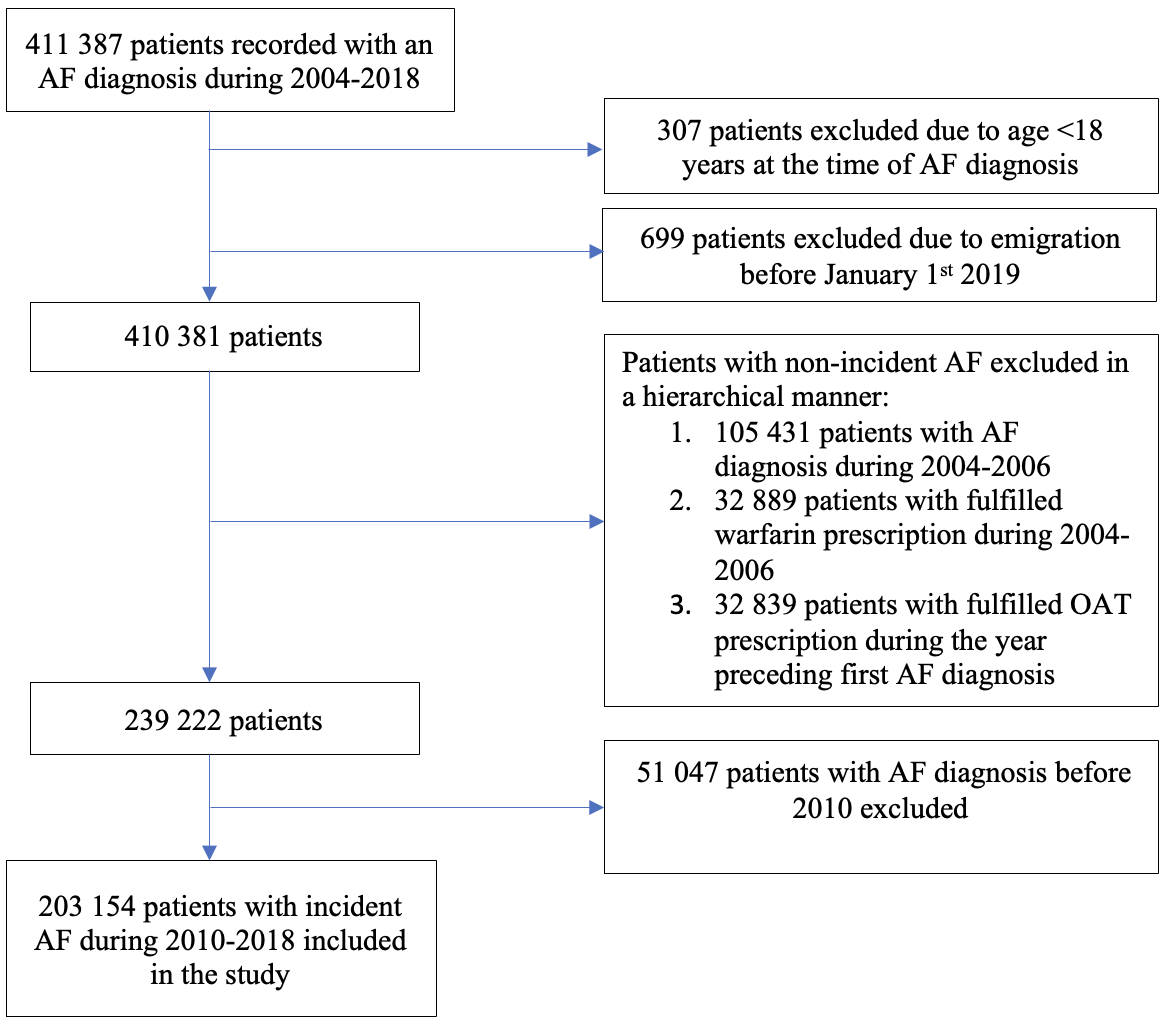


**Supplementary Figure 2.** Proportions of patients receiving AATs within one year follow-up according to the year of AF diagnosis

**
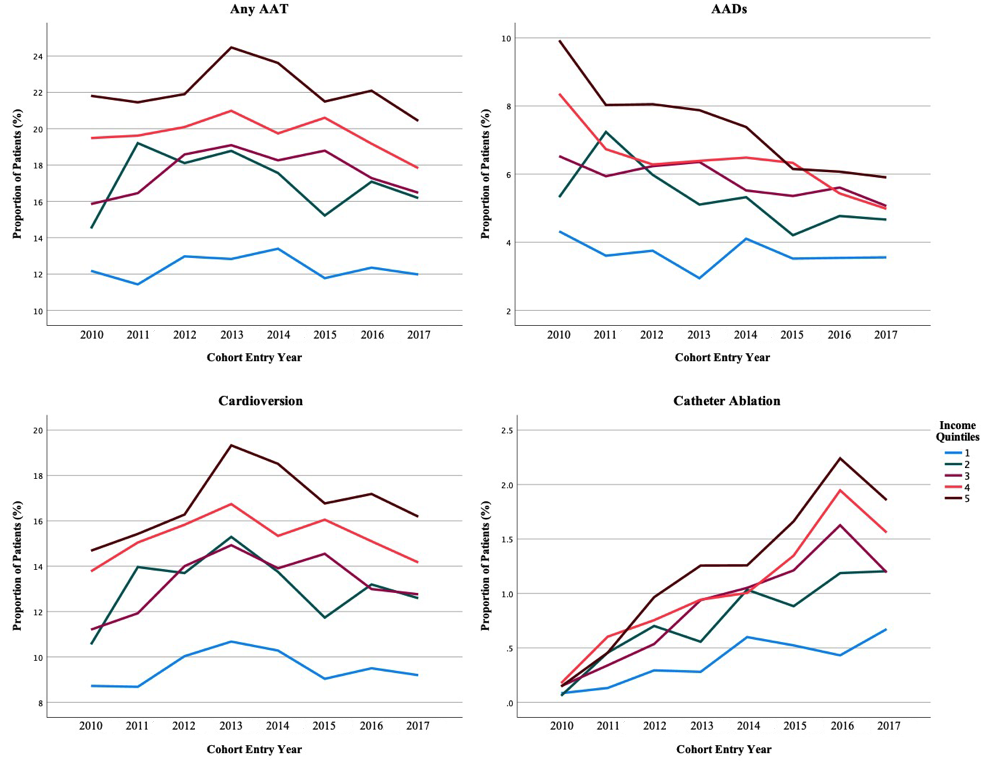
**

**Supplementary Figure 3.** Proportion of patients receiving any AAT within one year follow-up according to age at the time of AF diagnosis.

**
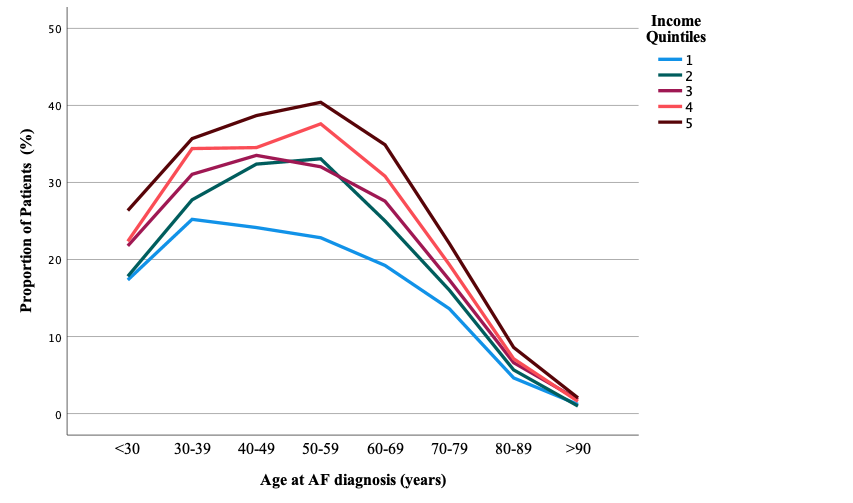
**
